# Cluster analysis of Model for End-stage Liver Disease and European System for Cardiac Operative Risk Evaluation for predicting operative mortality after concomitant tricuspid valve surgery

**DOI:** 10.1016/j.xjse.2024.100017

**Published:** 2024-08-05

**Authors:** Kevin Lim, Antony Chin Ching Au, Matthew Ming Hon Hui, Ivan Chi Hin Siu, Simon Chi Ying Chow, Jacky Yan Kit Ho, Song Wan, Randolph Hung Leung Wong

**Affiliations:** Division of Cardiothoracic Surgery, Prince of Wales Hospital, Hong Kong Special Administrative Region, China

**Keywords:** Model for End-stage Liver Disease, multiorgan failure, tricuspid valve surgery

## Abstract

**Objective:**

Patients requiring concomitant tricuspid surgery represent a heterogeneous cohort with significant comorbidities and varying degrees of organ and right ventricular dysfunction. However, surgeons can rely on little beyond intuition and experience when discussing operative risks. The objective of the study is to assess how the Model for End-stage Liver Disease score complements the European System for Cardiac Operative Risk Evaluation II in risk assessment.

**Methods:**

We performed a single-center retrospective cohort study of 369 consecutive patients who underwent concomitant tricuspid valve surgery from 2011 to 2020. Multivariate analysis of factors affecting operative mortality was performed, producing 2 multivariate risk prediction models, one consisting of European System for Cardiac Operative Risk Evaluation II components and the other consisting of both European System for Cardiac Operative Risk Evaluation II components and Model for End-stage Liver Disease. The models were compared by measuring c-statistic using the Hanley-McNeil method. This was further evaluated with category-free net reclassification improvement index. K-means clustering was performed using Model for End-stage Liver Disease and European System for Cardiac Operative Risk Evaluation II values after scalar transformation as independent variables and operative mortality as the dependent variable.

**Results:**

The Model for End-stage Liver Disease is an independent predictor of operative mortality, with an adjusted odds ratio of 1.286 per point. Inclusion of the Model for End-stage Liver Disease improves the discriminatory power of the European System for Cardiac Operative Risk Evaluation II for operative mortality, with a difference in area under the curve of 0.128 [0.0341-0.222] (*P* = .0076). The net reclassification improvement index of incorporating Model for End-stage Liver Disease with European System for Cardiac Operative Risk Evaluation II was 0.959 [0.515-1.392], indicating significant improvement in risk reclassification. Cluster analysis identified a unique cohort of patients with intermediate-to-high Model for End-stage Liver Disease, not previously identified with European System for Cardiac Operative Risk Evaluation II alone, who experienced high operative mortality.

**Conclusions:**

Model for End-stage Liver Disease score as a quantifier of hepatorenal dysfunction complements European System for Cardiac Operative Risk Evaluation II in predicting operative mortality after tricuspid valve surgery.

## Graphical Abstract

**Figure:**
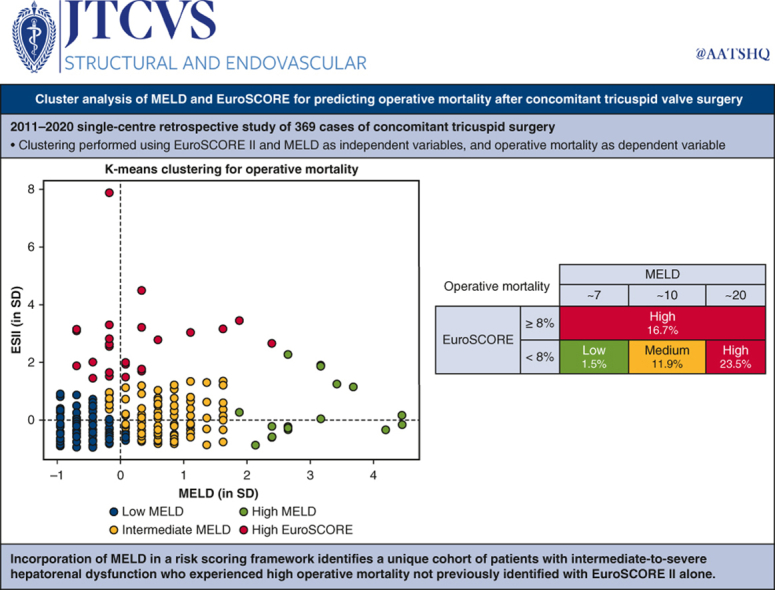

**Figure:**
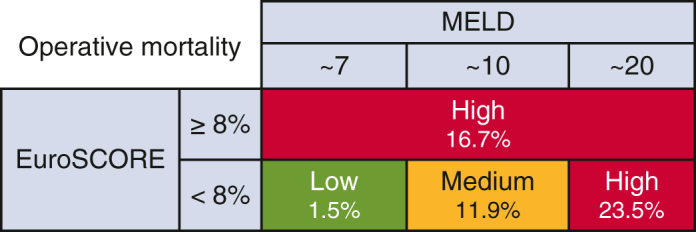
Matrix of EuroSCORE and MELD risk categories.

**Central Message**

The present article describes how MELD score as a quantifier of hepatorenal dysfunction aids in risk assessment of patients who require concomitant tricuspid valve surgery.

**Perspective**

Incorporation of MELD in a risk scoring framework identifies a unique cohort of patients with intermediate to severe hepatorenal dysfunction who experienced high operative mortality not previously identified with EuroSCORE II alone.

Functional tricuspid regurgitation (TR) as a sequelae of left heart disease is a morbid condition that, if left uncorrected at the index operation, is associated with subsequent right ventricular dysfunction, hepatic congestion, cardiac cirrhosis, and worsened survival.^1^, ^2^, ^3^

The importance of intervening on the tricuspid valve preemptively is increasingly recognized because of improved understanding of its natural history as well as advances in echocardiographic and intraoperative assessment.^4^^,^^5^ The increase in tricuspid operations recorded in the Society of Thoracic Surgeons Adult Cardiac Surgery Database reflected a paradigm shift over the past 2 decades toward preemptive prophylactic surgery at the time of left heart surgery.^6^

Despite its technical simplicity, the need for concomitant tricuspid valve surgery inherently implies an elevated risk of postoperative right ventricular failure and multiorgan dysfunction. The risk is proportional to the trifecta of preexisting right ventricular dysfunction, organ dysfunction, and comorbidities. Over the years, attempts to integrate and quantify these risk factors have produced various risk prediction models for isolated tricuspid valve surgery.^7^^,^^8^ More recently, the Society of Thoracic Surgeons has produced risk prediction models specifically for concomitant tricuspid valve surgery.

One of the most widely adopted models remains the Model for End-stage Liver Disease (MELD) score.^9^, ^10^, ^11^, ^12^, ^13^ In this article, we explore how incorporation of MELD in the European System for Cardiac Operative Risk Evaluation II (EuroSCORE II) framework aids the heart team in decision-making.

## Materials and Methods

### Study Design

This is a retrospective cohort study of 369 consecutive patients who underwent concomitant tricuspid valve surgery from January 1, 2011, to December 31, 2020, at the Prince of Wales Hospital. Two subjects were excluded because of concomitant pericardiectomy and ventricular septal rupture repair.

Our unit is a public tertiary referral center receiving referrals from 5 cardiology units in neighboring districts with a catchment population of 2.5 million Hong Kong residents. Historical electronic health records from 2005 onward can be accessed by both public and private healthcare practitioners on a need-to-know basis, irrespective of the location of healthcare provision within the city.

Baseline demographics, comorbidities, operative details, and postoperative complications and cardiac events were obtained by review of the electronic health records. EuroSCORE II definitions of risk factors were used. Patients with their follow-up more than 6 months before censorship were contacted by research personnel to verify survival status.

The study was authorized by the institutional research ethics board on June 9, 2022 (Reference Number: 2022.220). Consent was waived in view of its retrospective nature. Database lock date was June 30, 2022.

### Calculation of European System for Cardiac Operative Risk Evaluation II and Model for End-stage Liver Disease

EuroSCORE II predicted risk of mortality was calculated for all patients based on the original equation described by Nashef and colleagues.^14^ MELD 3.0 was used as the surrogate measure of the severity of hepatorenal dysfunction.^15^ The sodium, creatinine, bilirubin, and international normalized ratio (INR) measurements taken 1 day before surgery was used for calculating MELD. Pertaining to the accuracy of INR for patients on anticoagulants, 79% of the cohort had atrial fibrillation, and more than 90% of patients who had atrial fibrillation were on warfarin. The institutional protocol for perioperative warfarin management was cessation 4 days before surgery. As such, the last preoperative INR value likely reflected genuine hepatic function, more so than residual pharmacological effect.

### Definition of Operative Mortality and Perioperative Events

In adherence to the Valve Academic Research Consortium-2 consensus document, operative mortality was defined as follows: deaths from all causes within 30 days after discharge or deaths during the index procedure hospitalization if the postoperative stay is more than 30 days. Postoperative events were analyzed according to the guidelines for reporting morbidity and mortality after cardiac valvular operations. Acute liver failure was defined per the American Association for Study of Liver Diseases 2011 position paper as the presence of coagulation abnormalities, usually an INR greater than or equal to 1.5 and any degree of encephalopathy. Ancillary evidence included raised serum ammonia levels.

### Statistical Analysis

Descriptive statistics were reported as mean with SD or median with interquartile range for continuous variables and as frequencies and percentages for categorical variables. Difference between means was compared using Student *t* test after verifying equality of variances with Levene's test and normality of distribution with the Shapiro–Wilk test. Categorical variables were compared using the chi-square test when the minimum number of observations in a category was greater than 5; otherwise, likelihood ratio G-tests were used.

EuroSCORE II components were included in the multivariate analysis if the univariate association was significant to a *P* value less than .2 and removed using the backwards stepwise elimination procedure. Regression modeling was used to calculate the association between MELD and EuroSCORE II risk factors, producing 2 sets of predicted risk of mortality figures. The first regression model consisted of EuroSCORE II components only. The second regression model was constructed in a hierarchical fashion, consisting of EuroSCORE II components in the first layer and then introducing MELD in the second layer. The models were compared by measuring the c-statistic using the Hanley-McNeil method. This was further evaluated with category-free net reclassification improvement (NRI) index. The 95% CIs were estimated using 999 bootstrap replications. Hosmer–Lemeshow statistic was used to assess calibration across deciles of observed patient risk.

Cluster analysis was performed using actual EuroSCORE II values instead of the predicted risk of mortality figures from the previous analysis. Scalar transformation was performed for both EuroSCORE II and MELD to achieve data normalization. Clustering was performed using MELD and EuroSCORE II values as independent variables and operative mortality as the dependent variable. K-means clustering is an unsupervised machine learning technique that partitions data points into clusters based on similarity. The main objective of the algorithm is to minimize the sum of distances between the points and their respective cluster centroids. The ideal number of clusters was defined by elbow method. By plotting inertia (the sum of the distances of all the points within a cluster from the centroid of that cluster) against the number of clusters, the point at which decrease in inertia value becomes constant represents the right cluster value for the cohort. Clustering quality was assessed by silhouette score (measure of similarity of each point to its own cluster compared with other clusters). Operative mortality in each cluster was compared using the chi-square test.

A predetermined alpha value of 0.05 was used as the threshold for statistical significance. Bonferroni adjustment was applied to account for multiple comparisons. Statistical analysis and graphical design were performed with Python 3.11.

## Results

### Demographics and Baseline Characteristics

There were 194 men (53%) and 173 women (47%) in the cohort. Patients typically present late in the course of the disease, with half of the cohort experiencing New York Heart Association functional class III or IV symptoms at presentation. One-third of them had severe pulmonary hypertension, as defined by a mean pulmonary arterial pressure of greater than 55 mm Hg. Moderate-to-severe pulmonary hypertension was present in 90% of the cohort. Urgent operations due to refractory heart failure or repeated hospitalizations for heart failure accounted for 14% of all cases. Atrial fibrillation was present in 79%.

The indications for concomitant tricuspid valve surgery were as follows: severe TR (58.2%, n = 196), moderate TR (30.6%, n = 103), annular dilatation alone (10.1%, n = 34), and severe pulmonary hypertension alone (1.2%, n = 4).

### Operative Technique

All operations were performed through median sternotomy and aorto-bicaval cannulation. Cardioplegic arrest was achieved with antegrade cold blood crystalloid cardioplegia. Mitral valve surgery with concomitant tricuspid valve repair accounted for 88% of all operations. Degenerative and rheumatic pathologies each accounted for 40% of mitral valve operations. Mitral valve replacements accounted for 56% of all mitral valve operations, and the predominant pathology was rheumatic (70%). Overall, the repair rate for degenerative mitral regurgitation was 84%. Triple valve surgery was performed in 15% of patients, and 11% of all operations were reoperations.

The tricuspid valve was repaired in 93% of all cases; 92.6% received a remodeling annuloplasty, and 20% of all tricuspid operations were performed with the heart beating, at the discretion of the operating surgeon. The predominant pathologies were functional (53%) and endocarditis (33%).

### Operative Mortality

Operative mortality was 6.8%. Postoperative mechanical circulatory support was required in 7.1% of patients, of whom one-third required extracorporeal membrane oxygenation and the remainder required intra-aortic balloon counterpulsation. The mortality rate for patients who required mechanical circulatory support was 46%. The most common causes of operative mortality were low cardiac output syndrome (36%) and acute liver failure (28%); 5.2% of patients required resternotomy for secondary hemorrhage.

### Univariate and Multivariate Analyses

Comparison of baseline characteristics of survivors and non-survivors is shown in Table 1. Univariate analysis showed that those who died after the operation were more likely to be older and to have significant comorbidities, including extracardiac arteriopathy, immunocompromised state, and chronic kidney disease. The operations undertaken in non-survivors were more likely to be urgent in nature and of greater complexity (triple valve surgery and longer aortic crossclamp time).

**Table 1:** Comparison of demographics and baseline characteristics between survivors and non-survivors of the index operation

Variable	Overall	n (%) or mean ± SD or median [IQR]		Univariate analysis *P* value
		Survivors (n = 342)	Non-survivors (n = 25)	
Age (y)	61.6 ± 10.5	61.4 ± 10.7	65.4 ± 6.7	.009∗
Female sex	173 (47.1%)	161 (47.1%)	12 (48.0%)	1.000
Diabetes mellitus on insulin	13 (3.5%)	11 (20.0%)	2 (22.2%)	.878
Extracardiac arteriopathy	28 (7.6%)	20 (5.8%)	8 (32.0%)	.000002∗
Chronic pulmonary disease	54 (14.7%)	49 (14.3%)	5 (20.0%)	.391
Creatinine clearance (mL/min)				
<50	145 (39.5%)	130 (38.0%)	15 (62.5%)	.072∗
50-85	189 (51.5%)	176 (52.6%)	9 (36.0%)	
>85	27 (7.4%)	27 (7.9%)	0	
Dialysis required	6 (1.6%)	5 (1.5%)	1 (4.0%)	
Active endocarditis	18 (4.9%)	18 (5.3%)	0	.623
Immunocompromised state	4 (1.1%)	2 (0.6%)	2 (8.0%)	.025∗
Active or past intravenous drug use	7 (1.9%)	7 (2%)	0	1.000
Critical preoperative state	9 (2.5%)	8 (2.3%)	1 (4.0%)	.474
Poor mobility	4 (1.1%)	4 (1.2%)	0	1.000
NYHA				
Class I or II	183 (49.9%)	176 (51.5%)	7 (28.0%)	.019∗
Class III or IV	184 (50.1%)	166 (48.5%)	18 (72.0%)	
LV function				
LVEF ≥50%	297 (80.9%)	288 (81.0%)	20 (80.0%)	.762
LVEF 31%-49%	64 (17.4%)	59 (17.3%)	5 (20.0%)	
LVEF ≤30%	6 (1.6%)	6 (1.8%)	0	
Pulmonary hypertension				
None	41 (11.2%)	39 (11.4%)	2 (8.0%)	.677
Moderate 31-54 mm Hg	192 (52.3%)	180 (52.8%)	12 (48.0%)	
Severe ≥55 mm Hg	133 (36.2%)	122 (35.8%)	11 (44.0%)	
Liver disease		22 (6.4%)	1 (4.0%)	
Chronic hepatitis B	13 (3.5%)			1.000
Chronic hepatitis C	9 (2.5%)			
Primary biliary cirrhosis	1 (0.3%)			
Reoperation	39 (10.6%)	36 (10.5%)	3 (12.0%)	.510
Urgency				
Elective	311 (84.7%)	292 (85.4%)	19 (76.0%)	.126∗
Urgent	53 (14.4%)	48 (14.0%)	5 (20.0%)	
Emergency	3 (0.8%)	2 (0.6%)	1 (4.0%)	
Beating heart tricuspid operation	72 (19.6%)	68 (19.9%)	4 (16.0%)	.435
Tricuspid valve replacement	27 (7.4%)	24 (7.0%)	3 (12.0%)	.276
Bioprosthesis	20 (5.4%)			
Mechanical	7 (1.9%)			
Tricuspid pathology				
Functional	345 (94.0%)	322 (94.2%)	23 (92.0%)	.654
Nonfunctional		20 (5.8%)	2 (8.0%)	
Endocarditis	12 (3.3%)			
Rheumatic	6 (1.6%)			
Pacing lead	3 (0.8%)			
Radiotherapy	1 (0.3%)			
Concomitant surgery	39 (10.6%)	35 (10.2%)	4 (16.0%)	.322
CABG	13	13 (3.8%)	0	1.000
Aortic valve replacement	131	125 (36.5%)	6 (24.0%)	.280
Mitral valve repair	137	129 (37.7%)	8 (32.0%)	.671
Mitral valve replacement	56	47 (13.7%)	9 (36.0%)	.007∗
Triple valve surgery				
Thoracic aortic surgery	3 (0.8%)	2 (0.6%)	1 (4.0%)	.191∗
MELD	10.7 ± 4.7	10.4 ± 4.4	14.7 ± 6.2	.055∗
Cardiopulmonary bypass time (min)	156 ± 49	152 ± 41	216 ± 95	<.0001∗
Aortic crossclamp time (min)	103 ± 35	101 ± 28	136 ± 55	<.0001∗

*IQR*, Interquartile range; *NYHA*, New York Heart Association; *LV*, left ventricle; *LVEF*, left ventricular ejection fraction; *CABG*, coronary artery bypass grafting; *MELD*, Model for End-stage Liver Disease.

∗ *P* < .2, to be entered into multivariate model.

The first multivariate logistic regression model included risk factors found to be significant on univariate analysis with *P* value less than .2, including age, extracardiac arteriopathy, immunocompromised state, creatinine clearance, triple valve surgery, and crossclamp time. Results from this regression produced the first set of predicted risk of mortality figures.

The regression model was then re-run in a hierarchical fashion, introducing EuroSCORE risk factors in the first layer and MELD in the second layer. After controlling for age, extracardiac arteriopathy, and aortic crossclamp times, MELD continued to be an independent risk factor for operative mortality, with an adjusted odds ratio of 1.286 per MELD point (Table 2). Results from this regression produced the second set of predicted risk of mortality figures.

**Table 2:** Hierarchical logistic regression model for the association of MELD and EuroSCORE components with operative mortality

Operative mortality	AOR	
	AOR [95% CI]	*P* value
First layer – Significant variables from univariate analysis of EuroSCORE components		
Age	1.064 [1.000-1.128]	.038∗
Extracardiac arteriopathy	6.709 [2.094-21.495]	.001∗
NYHA class III and IV	0.517 [0.177-1.511]	.228
Urgency		
Urgent	0.531 [0.041-6.937]	.629
Emergency	0.759 [0.052-10.996]	.840
Triple valve surgery	0.673 [0.219-2.069]	.490
Aortic crossclamp time	1.025 [1.010-1.039]	.001∗
Second layer – Incorporated for the second multivariate model		
MELD	1.286 [1.108-1.493]	.001∗

*MELD*, Model for End-stage Liver Disease; *EuroSCORE*, European System for Cardiac Operative Risk Evaluation; *AOR*, adjusted odds ratio; *NYHA*, New York Heart Association.

∗ *P* < .05, statistically significant.

### Impact of Model for End-stage Liver Disease on Operative Mortality Risk Prediction: Discrimination, Calibration, and Reclassification

**Figure 1:**
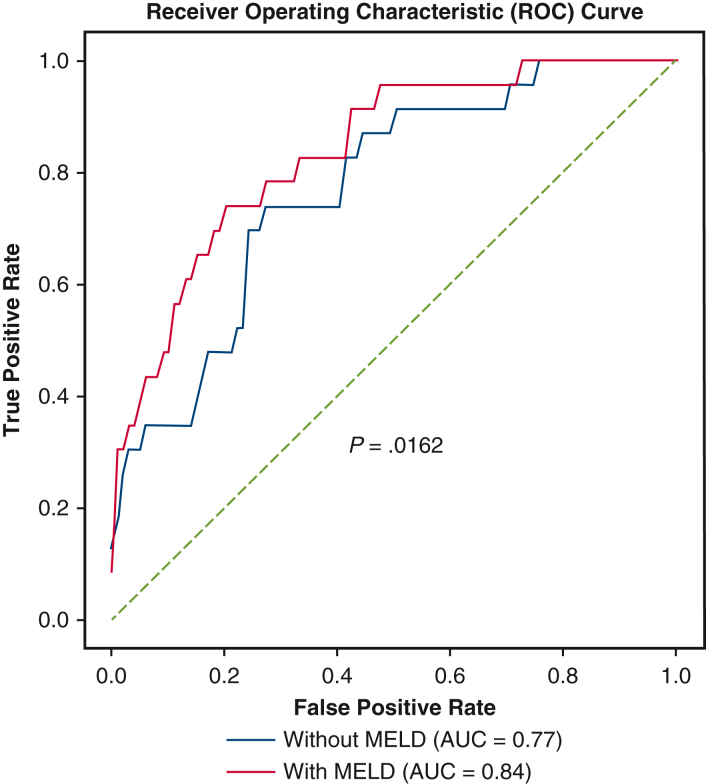
Receiver operating characteristic curves of the regression model with and without including MELD at prognosticating operative mortality after tricuspid valve surgery. Increase in discriminatory ability for operative mortality by inclusion of MELD. The *diagonal line* represents no discriminatory power (area under the receiver operating characteristic curve of 0.5). *Blue line*: without MELD. *Red line*: with MELD. *MELD*, Model for End-stage Liver Disease; *AUC*, area under the curve.

Using the 2 sets of predicted risk of mortality figures, the discriminatory ability of the regression model without and with MELD was compared using area under the receiver operating characteristic curves (Figure 1). The area under the curve without and with MELD were 0.715 [0.666-0.761] and 0.843 [0.802-0.879], respectively. Inclusion of MELD improves discriminatory power for operative mortality, using the Hanley-McNeil method (difference in area under the curve = 0.128 [0.0341-0.222], *P* = .0076). PseudoR^2^ showed that the regression model without MELD accounted for 23% of variance in outcomes, whereas the model with MELD accounted for 31.5% of variance in outcomes. Hosmer–Lemeshow test demonstrated that both models were calibrated for operative mortality (*P* = .225 and *P* = .347).

When assessed by the category-free net reclassification index, inclusion of MELD leads to a significant improvement in overall NRI of 0.959 [0.515-1.392] (Table 3). The event NRI is 0.392 [−0.03 to 0.809], which is not statistically significant. The nonevent NRI is 0.566 [0.460-0.657], which is statistically significant.

**Table 3:** Category-free net reclassification improvement contingency table for operative mortality

Operative mortality	n	Reclassified by MELD		NRI	
		Higher risk	Lower risk		
Survivor	342	104	238	Survivors	0.392 [–0.037-0.809]
Non-survivors	25	21	4	Non-survivors	0.566 [0.460-0.657]
Overall category-free NRI _>__0_ [95% bootstrap CI]					0.959 [0.515-1.392]

*MELD*, Model for End-stage Liver Disease; *NRI*, net reclassification improvement.

### Cluster Analysis

**Figure 2:**
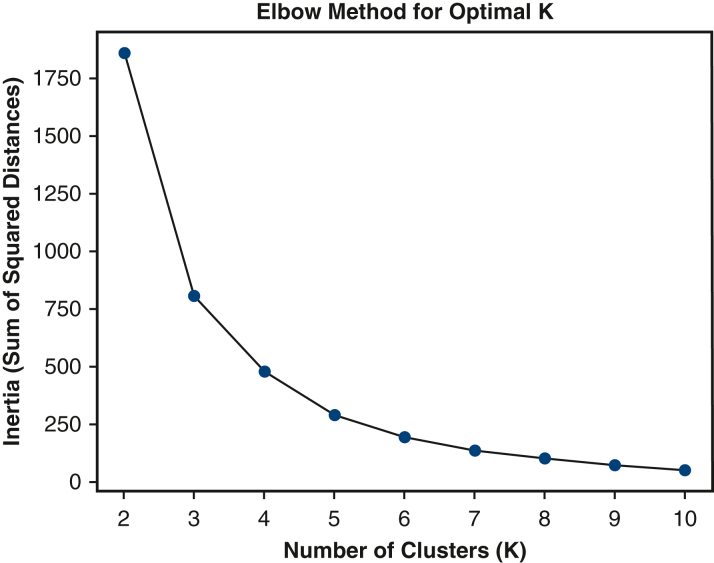
Elbow method for identifying the optimal number of clusters. By plotting inertia against the number of clusters, the point at which decrease in inertia value becomes constant represents the right cluster value for the cohort.

**Figure 3:**
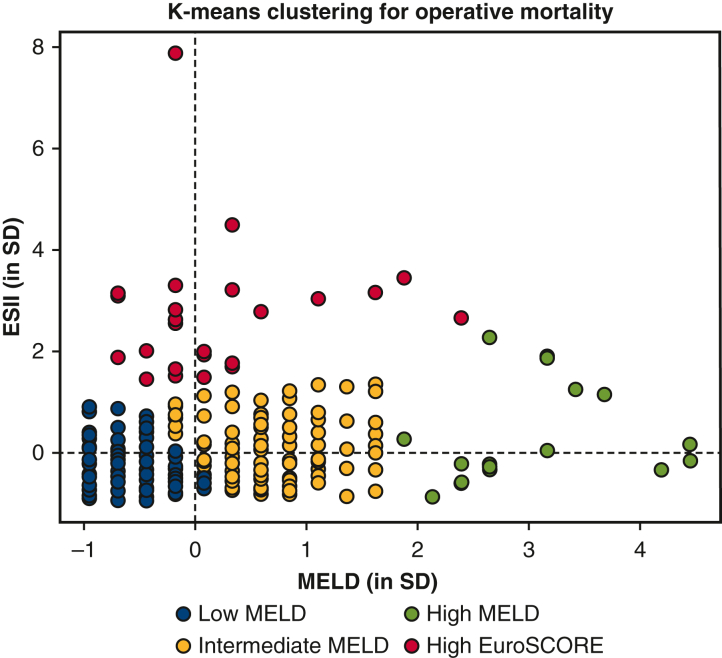
K-means cluster analysis of MELD, EuroSCORE II, and operative mortality. The matrix identifies unique subgroups of patients with intermediate-to-high MELD who are at elevated risk of operative mortality. *MELD*, Model for End-stage Liver Disease; *EuroSCORE*, European System for Cardiac Operative Risk Evaluation; *ESII*, European System for Cardiac Operative Risk Evaluation II.

Cluster analysis was performed using actual EuroSCORE II values instead of the predicted risk of mortality figures from the previous analysis. The clustering algorithm was iterated multiple times to determine the ideal number of clusters. By using the elbow method, the ideal number of clusters was 4 (Figure 2). A scatterplot of EuroSCORE II and MELD as well as the 4 clusters produced are shown in Figure 3. The silhouette score was 0.4538, indicating adequate cluster separation.

The characteristics of the 4 clusters and the corresponding operative mortality were as follows:

- Cluster 1: Low MELD (mean ± SD: 7.32 ± 1.20): 1.5% (n = 195)
- Cluster 2: Intermediate MELD (mean ± SD: 12.12 ± 1.89): 11.9% (n = 101)
- Cluster 3: High MELD (mean ± SD: 21.47 ± 3.06): 23.5% (n = 17)
- Cluster 4: High EuroSCORE II (mean ± SD: 17.8 ± 6.2%): 16.7% (n = 24)

The chi-square test showed a statistically significant difference in operative mortality between cluster 1 and other clusters. However, there was no statistically significant difference in operative mortality among clusters 2, 3, and 4.

**Figure 4:**
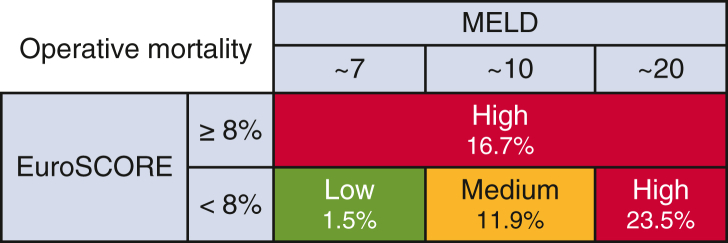
Matrix of EuroSCORE II and MELD risk categories. Unique clusters of patients with intermediate-to-high MELD scores with low EuroSCORE II had high operative mortality. *EuroSCORE*, European System for Cardiac Operative Risk Evaluation; *MELD*, Model for End-stage Liver Disease.

With the cluster analysis as the conceptual framework, a risk matrix can be created by cross-tabulation of EuroSCORE II and MELD scores to create a risk matrix (Figure 4). This matrix identifies 4 subgroups of patients with different risk strata for concomitant tricuspid valve surgery, with the lowest risk group having adequate organ and cardiac reserve and the highest risk group having severe organ or cardiac dysfunction. This conceptual framework is currently in use in our institutional heart team discussions.

## Discussion

**Figure 5:**
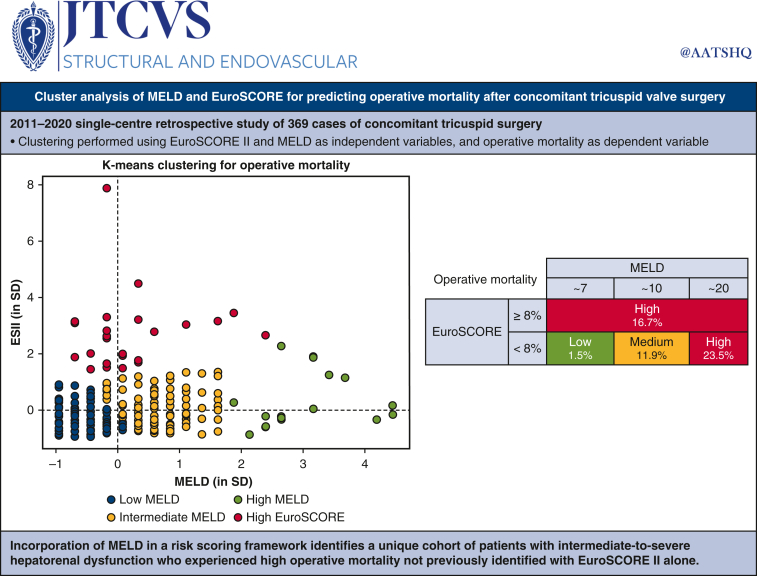
Cluster analysis of MELD and EuroSCORE for predicting operative mortality after concomitant tricuspid valve surgery. *MELD*, Model for End-stage Liver Disease; *EuroSCORE*, European System for Cardiac Operative Risk Evaluation.

The goal of this retrospective cohort study is to assess the utility of a model using EuroSCORE II and MELD for risk assessment for concomitant tricuspid valve surgery. There are 2 key findings. First, in the cluster analysis, incorporation of MELD identifies a unique cohort of patients with intermediate-to-severe hepatorenal dysfunction who experienced high operative mortality not previously identified with EuroSCORE II alone. Second, in the reclassification analysis, the nonevent NRI is highly significant, but the event NRI is not. The implication is that the new risk model primarily works by reclassifying high-risk individuals who survive the operation into a lower-risk tier. Figure 5 shows a graphical abstract of the study.

The risk matrix generated from the cluster analysis can aid the heart team in identifying 2 patient subgroups who are at elevated risk of operative mortality after concomitant tricuspid surgery. The first subgroup (Figure 3, intermediate MELD) has poor organ reserve, and thus may benefit from aggressive preoperative optimization and preemptive intraoperative mechanical circulatory support. At our institution, these cases are undertaken by 2 specialist valve surgeons to smooth operation flow and reduce pump time. The second subgroup (Figure 3, high MELD) usually has both severe organ dysfunction and multiple comorbidities and cannot tolerate conventional surgery. In heart team discussions, these patients are typically only considered for transcatheter therapies.

The Society of Thoracic Surgeons currently provides 2 models for risk assessment of concomitant tricuspid surgery, which can readily calculate predicted risk of mortality figures. The contemporary STS Adult Cardiac Surgery Database calculator is calibrated for concomitant tricuspid valve repair in the context of a mitral procedure but does not incorporate MELD subcomponents. The Multi-Valve Surgery Risk Calculator incorporates MELD subcomponents but is only calibrated for concomitant tricuspid repair in the context of a double-valve procedure. These limitations reduce their applicability to patients with long-standing TR, whose mortality is driven primarily by right heart failure and organ dysfunction. Despite statistical sophistication and continuous refinement, decision-making in multivalvular surgery continues to rely heavily on intuition and experience.

In 1976, British statistician George Box wrote the famous line, “All models are wrong, some are useful.”^16^ In developing a risk model, the philosophical concept of parsimony should be adhered to.^17^ The concept of parsimony states that the simplest explanation of a phenomenon is often the preferred one. If a simple model can explain a phenomenon with the same level of precision as more complex models, the simpler model should be preferred.^18^ We believe that the risk model presented here fulfils this test.

### Limitations

The study has limitations inherent to all retrospective studies, including the inability to prove causation and selection bias. The sample size is small relative to the scale of national and international databases. Conclusions drawn would require external validation. Perioperative hemodynamic data were not routinely recorded and thus absent from multivariate analysis.

Right ventricle dysfunction is a missing piece of the puzzle and a key area for future investigation. The effect of right ventricular systolic dysfunction on operative mortality has been poorly quantified, because right ventricular function itself is difficult to quantify. The use of novel echocardiographic parameters including right ventricular strain and right ventricular-pulmonary arterial coupling may allow objective quantification of right ventricular function.^19^ Further studies in this area are eagerly awaited and may improve the nuances of decision-making for tricuspid surgery.

## Conclusions

The MELD score as a quantifier of hepatorenal dysfunction complements the EuroSCORE II in discriminating and reclassifying patients at high risk of operative mortality and long-term events after tricuspid valve surgery.

## Conflict of Interest Statement

The authors reported no conflicts of interest.

The *Journal* policy requires editors and reviewers to disclose conflicts of interest and to decline handling or reviewing manuscripts for which they may have a conflict of interest. The editors and reviewers of this article have no conflicts of interest.
